# The good and the bad about separation anxiety: roles of IL-22 and IL-22BP in liver pathologies

**DOI:** 10.1007/s00281-021-00854-z

**Published:** 2021-04-13

**Authors:** Jöran Lücke, Morsal Sabihi, Tao Zhang, Lennart Fynn Bauditz, Ahmad Mustafa Shiri, Anastasios D. Giannou, Samuel Huber

**Affiliations:** 1 Section of Molecular Immunology und Gastroenterology, I. Department of Medicine, University Medical Center Hamburg-Eppendorf, 20246 Hamburg, Germany; 2 Hamburg Center for Translational Immunology (HCTI), University Medical Center Hamburg-Eppendorf, 20246 Hamburg, Germany; 3 Department of General, Visceral and Thoracic Surgery, University Medical Center Hamburg-Eppendorf, 20246 Hamburg, Germany

**Keywords:** Hepatocellular carcinoma, Liver damage, Liver infection, IL-22, IL-22BP

## Abstract

The human liver fulfills several vital tasks daily and possesses an impressive ability to self-regenerate. However, the capacity of this self-healing process can be exhausted by a variety of different liver diseases, such as alcoholic liver damage, viral hepatitis, or hepatocellular carcinoma. Over time, all these diseases generally lead to progressive liver failure that can become fatal if left untreated. Thus, a great effort has been directed towards the development of innovative therapies. The most recently discovered therapies often involve modifying the patient’s immune system to enhance a beneficial immune response. Current data suggest that, among others, the cytokine IL-22 might be a promising therapeutical candidate. IL-22 and its endogenous antagonist, IL-22BP, have been under thorough scientific investigation for nearly 20 years. While IL-22 is mainly produced by T_H_22 cells, ILC3s, NKT cells, or γδ T cells, sources of IL-22BP include dendritic cells, eosinophils, and CD4^+^ cells. In many settings, IL-22 was shown to promote regenerative potential and, thus, could protect tissues from pathogens and damage. However, the effects of IL-22 during carcinogenesis are more ambiguous and depend on the tumor entity and microenvironment. In line with its capabilities of neutralizing IL-22 in vivo, IL-22BP possesses often, but not always, an inverse expression pattern compared to its ligand. In this comprehensive review, we will summarize past and current findings regarding the roles of IL-22 and IL-22BP in liver diseases with a particular focus on the leading causes of advanced liver failure, namely, liver infections, liver damage, and liver malignancies.

## Introduction

### IL-22 and IL-22BP

Separation anxiety mainly manifests in toddlers and smaller children and is regarded as a perfectly physiological behavior up to a certain point [1]. It can be defined as an unspecific fear of situations in which children are forcefully divided from their parents for a longer period of time [2]. However, once children grow older, such a fear of separation and abandonment is viewed as more critical, and is then classified as a separation anxiety disorder [1]. In these cases, patients fear abandonment by attachment figures, and thus often attempt to prevent such a suspected separation [2]. Likewise, plenty of molecular structures in our bodies are incapable of letting go, too. In the broad field of immunology, binding proteins (BP) for interleukins (IL) are a prominent example, exhibiting a high binding affinity for their specific targets. On the one hand, a strong bond between target and binding protein is often regarded as a valuable regulatory mechanism since excessive amounts of protein can be neutralized by the specific BP [3]. On the other hand, BPs can interfere with interleukin-mediated effects and dampen a potentially protective immune response [3]. Thus, tight spatiotemporal regulation of BPs is generally required to maximize their beneficial effects. Research of the last decade has led to comprehensive insight into the two known immunological pairings, namely, IL-18/IL-18BP [4] and IL-22/IL-22BP [5].

The cytokine IL-22 was discovered in 2000 and was named interleukin-10-related T cell-derived inducible factor (IL-TIF) at first [6]. According to its initial name, IL-22 indeed displays a high homology to IL-10 and is thus grouped into the IL-10 family of cytokines [7]. IL-22 can be produced by various immune cells, highly dependent on the examined tissue and present disease. Among these cell subsets, T helper 17 (T_H_17) cells [8–10], T helper 22 (T_H_22) cells [11], type 3 innate lymphoid cells (ILC3s) [12, 13], natural killer T (NKT) cells [14], and γδ T cells [9, 13, 15] comprise the most common IL-22 producers. Downstream signaling of this cytokine requires the binding to its heterodimeric receptor, consisting of IL-10R2 and IL-22RA1 [16]. While IL-10R2 is universally expressed and is involved in signal transduction of many cytokines of the IL-10-family, IL-22RA1 exclusively binds to IL-22 [7]. Moreover, the expression of IL-22RA1 is restricted to non-hematopoietic cells [17]. This marks IL-22 as one of the few interleukins that, contrarily to the original meaning of the word (“inter” = between [Latin], “leukos” =white [Greek]), actually do not mediate communication between leucocytes. Downstream of its receptor, effects of IL-22-signaling are mediated by activation of signal transducer and activator of transcription (STAT) 3, although STAT1 and STAT5 can be equally phosphorylated in hepatoma cells upon IL-22-stimulation [18]. Moreover, an IL-22-mediated activation of STAT1 also occurs in synergy with different types of interferons. For example, interferon-lambda (IFNλ) can mediate a STAT1-dependent protection from rotavirus infection in alliance with IL-22 [19], whereas interferon-alpha (IFNα) and IL-22 may conspire to aggravate graft versus host disease via STAT1 [20].

The general functions of IL-22 can be divided into two main pillars, classifying IL-22 as an anti-infectious and mainly pro-inflammatory cytokine. Firstly, IL-22 plays a pivotal role at barrier sites such as the intestinal tract and the skin, protecting the host from external infectious threats [21]. Secondly, IL-22 enhances tissue regeneration and wound healing in multiple tissues [22]. However, overt or insufficiently controlled IL-22 can promote inflammatory diseases and malignant progression in many organs by hijacking otherwise physiological pathways [23, 24]. Thus, tight control of IL-22 on different levels seems necessary to limit its pathological effects.

As mentioned above, the soluble receptor IL-22BP can bind and neutralize IL-22. Although it shares a highly similar protein structure to IL-22RA1 [25], IL-22BP possesses a much higher binding affinity to IL-22 than its membrane-bound equivalent [25]. Once bound to IL-22, IL-22BP can successfully block and neutralize the activity of IL-22 in vitro [26–28] and in vivo [5] by decreasing its availability for IL-22RA1. IL-22BP has been reported to be produced by different cell types, among them are dendritic cells (DC) [5, 29], eosinophils [29, 30], and cluster of differentiation (CD)4^+^ T cells [29, 31]. Generally, an inverse relation between IL-22BP and IL-22 can be observed in many tissues [5, 21, 32]. In physiological environments, IL-22BP is often highly expressed, keeping overt production of IL-22 in check [5, 21, 32]. After tissue damage, or during infections and subsequent inflammation, IL-22BP is downregulated by caspase-1-mediated activation of IL-18 [5, 32]. Reduced amounts of IL-22BP then allow IL-22 to exert its physiologically intended functions. Consequently, IL-22BP is found to be downregulated in autoinflammatory conditions such as psoriasis [33], but also in malignant diseases [29]. Low levels of IL-22BP then enhance a dysregulated IL-22-production promoting the progress of the disease. However, in some diseases like inflammatory bowel disease, IL-22BP can be found upregulated simultaneously to its ligand [31], suggesting that high levels of IL-22 can partially overrule an increased expression of IL-22BP.

### The liver and its pathologies

The liver is a vital organ responsible for orchestrating different metabolic pathways within the host. It is not only involved in degrading carbohydrates, proteins, and fat but is also pivotal for the storage of glycogen and the detoxification of many metabolic substrates [34]. On a structural level, the liver profits from a doubled blood supply with nutritious venous blood from the portal vein and blood with a high oxygen-saturation from the hepatic arteria. The blood of the two sources merges into one combined liver sinusoid that is confined by fenestrated epithelial cells. These cells are also termed liver sinusoidal endothelial cells (LSECs) [34]. The LSECs themselves are surrounded by a row of hepatocytes, only divided by a fine hemline, referred to as the space of Disse. Nutrients and toxic substances can easily penetrate the fenestrated LSECs, diffuse through the space of Disse, and are then absorbed by the hepatocytes. On the other side, facing away from the LSECs, hepatocytes secrete their produced substrates in the canal of Herring, which merges with other bile canaliculi to the bile duct [34]. Finally, the bile duct and the pancreatic duct drain into the duodenum.

Liver pathologies can be broadly separated into three main clusters: infections, damage, and malignant transformation. Infections of liver tissue can occur by various pathogens, including bacteria, viruses, protozoa, and helminths. The liver can either be affected by systemic infections that co-affect the liver, such as malaria [35], or by infectious pathogens that are capable of specifically targeting the liver, such as hepatitis B virus (HBV) and hepatitis C virus (HCV) [36]. Chronic infections lead to the second main entity of liver disease, namely, liver-specific tissue damage. Moreover, chronic liver damage and chronic infections can trigger liver cirrhosis by causing a dysregulated inflammatory response [37]. As well as recurring or persistent liver infections, other triggers for chronic liver damage exist, such as a high-fat diet or overt consumption of alcoholic beverages, resulting in nonalcoholic steatohepatitis (NASH) or alcoholic liver disease (ALD), respectively [37]. Furthermore, progressing liver cirrhosis is a potentially life-threatening disease with currently limited therapeutic options [37].

The third big cluster of liver diseases consists of liver malignancies. While hepatocellular carcinoma (HCC) most commonly arises due to pre-existing liver cirrhosis [38], the etiology and causes of other liver malignancies in the liver, such as cholangiocarcinoma (CCA), have only partially been identified [39]. An extension of liver malignancies comprises of liver metastasis, describing the seeding of a tumor derived from a distinct origin into the liver. These three described malignant entities are often diagnosed in a rather late stage due to the lack of specific symptoms in earlier stages. The only routinely performed screening-protocol refers to regular sonography of patients suffering from liver cirrhosis to detect early indicators of HCC development [40]. Thus, further diagnostic and therapeutic approaches to detect and treat liver disease, especially liver malignancies, are urgently warranted.

## IL-22 and IL-22BP in liver infections

### Commensal microbiota

The human gastrointestinal tract is the home of billions of different bacterial species. While the host provides them with nutrients, the commensal microbiota helps the body decompose otherwise indigestible food components, educates the adaptive immune system, and represses the expansion of potential pathobionts and pathogens. However, strict control of epithelial integrity and prevention of bacterial overgrowth are needed to inhibit pathological conditions such as dysbiosis [41]. IL-22 is considered a critical mediator of these particular features, ultimately enabling homeostasis maintenance in the gastrointestinal tract.

Gut-derived microbiota has also been shown to play an important role in regulating processes at extra-intestinal sites, an example being in the liver. The gut and liver can influence each other via a strong communication channel, termed the gut-liver-axis. Apart from the microbiome, mediators of this communication method also include immune cells, inflammatory molecules, and bile acids. Many ailments affecting the gut and the liver have often been associated with perturbations to the host microbiota. As well as allowing translocation of pathogenic or opportunistic microbial species, dysbiosis can induce the release of toxic microbial metabolites and inflammatory host factors that can cause liver injury when dysregulated [42]. Thus, the protective effects of IL-22 in the gut are equally important for maintaining homeostasis in the liver.

Moreover, microbiota-derived factors have also been shown to impact the direct effects of IL-22 and affect the overall inflammatory process within the hepatic tissue itself. In the liver, IL-22 can induce the production of many different antimicrobial peptides, all of which have different mechanisms of action, protecting the host from variable pathogens. Furthermore, this implies that many liver diseases exert not only direct effects on the levels of IL-22 in liver tissues but also imply indirect effects on IL-22 production by influencing the host microbiome.

Particularly, diseases such as NASH and ALD are closely associated with changes in host-microbiota composition. Alterations to the gut microbiota composition may arise due to high caloric intake, alcohol consumption, substance abuse, and general lifestyle changes. However, the mechanisms behind these have not been completely elucidated. Interestingly, a few recent studies have identified specific microbial factors that are either essential to alleviating these liver diseases or can be removed to improve the overall outcomes. Likewise, IL-22 can be influenced due to a modulation of the intestinal microbiome by these liver diseases [43–45].

For example, in patients with ALD, ethanol-induced changes to the host’s microbial composition result in lower levels of IL-22 production in the gut [43]. This was attributed to the depletion of the microbial species *Roseburia intestinalis*. Its absence in the host resulted in decreased levels of IL-22 and increased production of regenerating islet-derived protein 3 gamma (Reg3g) [43]. Another study showed that ethanol consumption diminished microbiota-derived ligands of the aryl hydrocarbon receptor (AhR), leading to decreased IL-22 and Reg3g production [44]. The authors of this study were also able to restore IL-22 levels and diminish ethanol-induced steatohepatitis by feeding the mice with genetically engineered IL-22-producing *Lactobacillus reuteri* [44]. Lately, it was found that an increased abundance of beneficial bacteria, such as *Lactobacillus* and *Christenellaceae*, enhanced a protective production of IL-22, which exerted particularly anti-inflammatory properties in NASH patients [45]. To summarize, liver disease often leads to a shift in the commensal microbiome, inducing decreased levels of IL-22. Since IL-22 is considered to be mostly protective in these settings, this described mechanism might further aggravate liver pathologies. However, IL-22 also plays a pivotal role in direct infections of the liver by many different bacteria, protozoa, helminths, and viruses. The contribution of this cytokine to these infectious diseases will be discussed in the following section of this review.

### Bacterial infections

A bacterial entity known for affecting the liver is *Listeria monocytogenes*, a particular bacterial species that can infect hepatocytes intracellularly. At least two studies observing cytokine production during *Listeria monocytogenes* infection describe an upregulation of IL-22 [46, 47]. Mechanistically, one of these studies found that IL-22 produced by CD4^+^ T cells was able to induce the production of the antimicrobial peptide phospholipase group II A (PLA2G2A) in hepatocytes [47]. In turn, PLA2G2A was shown to suppress infection of HepG2 cells in vitro at an early time point of *Listeria monocytogenes* infection [47]. Another pathogen commonly affecting the liver is *Klebsiella pneumoniae*. In this case, IL-22 can significantly reduce infection by this pathogen via induction of other antimicrobial peptides such as lipocalin 2 (LCN2) and serum amyloid A1 and A2 (SAA1/2). One study showed that the bacterial burden and the areas of liver necrosis were significantly reduced when either administering recombinant IL-22 or overexpressing IL-22 in a mouse model of acute peritoneal *Klebsiella pneumoniae* infection. Additionally, the presence of IL-22 also had a great impact on the overall survival rates of these mice, in the sense that IL-22 transgenic (IL-22TG) mice survived much longer than their wild-type counterparts [48]. Taken together, mounting evidence points towards a protective effect of IL-22 in bacterial liver infection.

### Protozoan infections

So far, studies investigating the effect of IL-22 on protozoan infections have mainly been based on *Plasmodium* infections. This class of pathobionts is known to cause malaria and can affect many organs in the host, including the liver. An involvement of IL-22 in influencing malaria infection was suspected early on after distinct gene polymorphisms related to the transcription of this cytokine were identified in West African patients. These single nucleotide polymorphisms (SNPs) described rendered the patients either more resistant or more susceptible to malaria infection [49]. Similarly, other IL-22-related SNPs identified in different ethnicities have emerged over the years [50, 51]. In line with the previous finding, elevated levels of IL-22 have been described in patients infected with *Plasmodium* species [52, 53]. Among these reports, it was discovered that polymorphisms in IL-22 were equally associated with a predisposition for childhood cerebral malaria [51]. One of the SNPs that the authors of this study identified was previously shown to induce higher production of IL-22 in CD4^+^ T cells ex vivo and higher binding efficiency of its transcription factor, AhR [54]. Interestingly, the authors failed to find an association between cerebral malaria and IL-22BP, indicating that IL-22BP may not be available to regulate IL-22 and its pro-inflammatory effects in these children [51].

In mouse models studying the inflammatory reaction caused by *Plasmodium* species, IL-22 has been shown to generally exert protective effects. Authors of one study found that IL-22 is predominantly produced by CD8^+^ T cells in the liver and that IL-22 is critical in protecting against liver damage. Ultimately, the lack of IL-22 resulted in a mortality rate of 50% in *Plasmodium chabaudi*-infected mice [55]. Another study describing infections with a different *Plasmodium* strain, namely, *Plasmodium berghei ANKA*, demonstrated a critical role of IL-22 in this infectious setting, as IL-22-deficient mice had a more severe disease outcome. Here, it was shown that IL-22 is mainly produced by γδ T cells and that the absence of IL-22 resulted in an earlier occurrence of cerebral malaria, although these mice exhibited a lower parasitemia. Interestingly, higher levels of interferon-gamma (IFNγ) were observed in IL-22-deficient mice. This indicates that IL-22 is either capable of dampening the IFNγ-response in these mice or that IFNγ gets upregulated as a compensatory mechanism in IL-22-deficient mice. However, the benefits or disadvantages of this observation cannot be determined without further investigation [53].

Lastly, *Plasmodium* co-infection with other pathogenic entities, such as helminths and viruses, is common and can be extremely lethal. To date, not much is known about immune regulation and the disease progression of any of these infections in humans; therefore, animal models have been utilized to understand these aspects better. In 2009, authors studied the coinfection of *Plasmodium chabaudi* and the nematode *Heligmosomoides bakeri polygyrus* in mice. These mice developed not only severe liver pathology but also portrayed a significant upregulation of IFNγ, IL-17A, and IL-22 expression in the liver [56]. Another study focused on understanding the immune reaction of a co-infection with *Plasmodium fragile* and simian-human immunodeficiency virus (SHIV) in rhesus macaques. It was shown that malaria co-infection of acutely SHIV-infected macaques led to high parasitemia, fatal virus-associated malaria, and accelerating SHIV disease. In chronically SHIV-infected macaques co-infected with *Plasmodium fragile*, a suppression of T_H_1 cells producing IFNγ could be observed, whereas IL-17A and IL-22 secretion from CD4^+^ and CD8^+^ T cells were greatly upregulated [57]. In summary, an increase in IL-22 production could be observed in protozoan infections as it could be in co-infections with other pathobionts. In particular, the function of IL-22 in malaria infections needs to be further elucidated, as this cytokine was obviously demonstrated to have a great impact on liver pathology and the overall survival of the host.

### Helminth infections

Typically, helminths cause long-lived infections that induce a more subdued or even suppressive immune response. Multiple attempts have been made to understand the association between helminth infections and IL-22 as a central mediator of the host’s immune response in the gut and the liver. In one study, it was found that schistosome eggs cultured with blood drawn from patients chronically infected with *Schistosoma japonicum* resulted in elevated IL-22 transcript levels, whereas IL-22BP transcripts were diminished. Furthermore, this study implied a protective role of IL-22 in this setting, as patients with high levels of this cytokine presented decreased hepatic fibrosis and portal hypertension. Contrastingly, the authors could identify specific SNP genotypes of IL-22BP that correlated with severe fibrosis, leading to the assumption that IL-22BP may be pathogenic in infections with *Schistosoma japonicum*. Strikingly, the same SNPs of IL-22BP were equally identified in hepatitis C patients and were yet again associated with hepatic fibrosis [58].

Another common helminth, the liver fluke *Opisthorchis viverrini*, is strongly associated with cholangiocarcinoma (CCA) development, particularly in Asian regions. One study provided evidence that liver fluke infections can induce significantly higher IL-17A and IL-22 expression levels than healthy controls and CCA patients without an infection [59]. Thus, it can be inferred that inflammation associated with *Opisthorchis viverrini* characterized by high levels of IL-17A and IL-22 may contribute to CCA development [59]. However, further studies are needed to determine the role of IL-22 in helminth infections.

### Viral infections

Intriguingly, the effects of IL-22 during viral infections in the liver seem to be dual-natured. Furthermore, they highly depend on the entity of the pathogenic virus, the duration, and the severity of liver inflammation. The protective effects of IL-22 in virus infections are exemplified in a study analyzing the impact of IL-22 in a setting of dengue infection [60]. Here, it was shown that infection with Dengue virus 2 (DENV2) in mice resulted in an upregulated expression of IL-22 and IL-17A. Significantly, IL-22-deficient mice developed a more severe infection, resulting in a higher mortality rate, an increased viral titer and exacerbated liver injury than their wild-type counterparts. Moreover, it was shown that IL-22 derived from NK and CD4^+^ T cells exerted protective functions in this experimental viral setting, whereas IL-17A derived from γδ T cells contributed to pathogenic inflammation [60].

Determining the effects and function of IL-22 in chronic viral infections such as HBV and HCV infections has been proven very difficult so far [61]. Until now, many studies point towards divergent roles of this cytokine; however, the majority report an increase in IL-22 production in HBV and HCV patients [61–64]. Interestingly, it has been revealed that IL-22 itself has only a minimal direct antiviral effect [61–64]. On the one hand, a protective trait of IL-22 has been identified in chronic HBV-infected patients, where T cell-derived IL-22 promoted hepatocyte survival and proliferation of liver progenitor cells [65]. On the other hand, the detrimental effects of IL-22 have equally been identified in chronic infections caused by both HBV and HCV. Polymorphisms in IL-22 are likewise implicated in HCV viral clearance and treatment response [66]. In HBV- and HCV- infected patients, IL-22 was positively correlated with liver fibrosis stages [67, 68]. Furthermore, two independent studies found that neutralization of IL-22 in transgenic HBV mice resulted in ameliorated liver inflammation and fibrosis [67, 69].

**Fig. 1:**
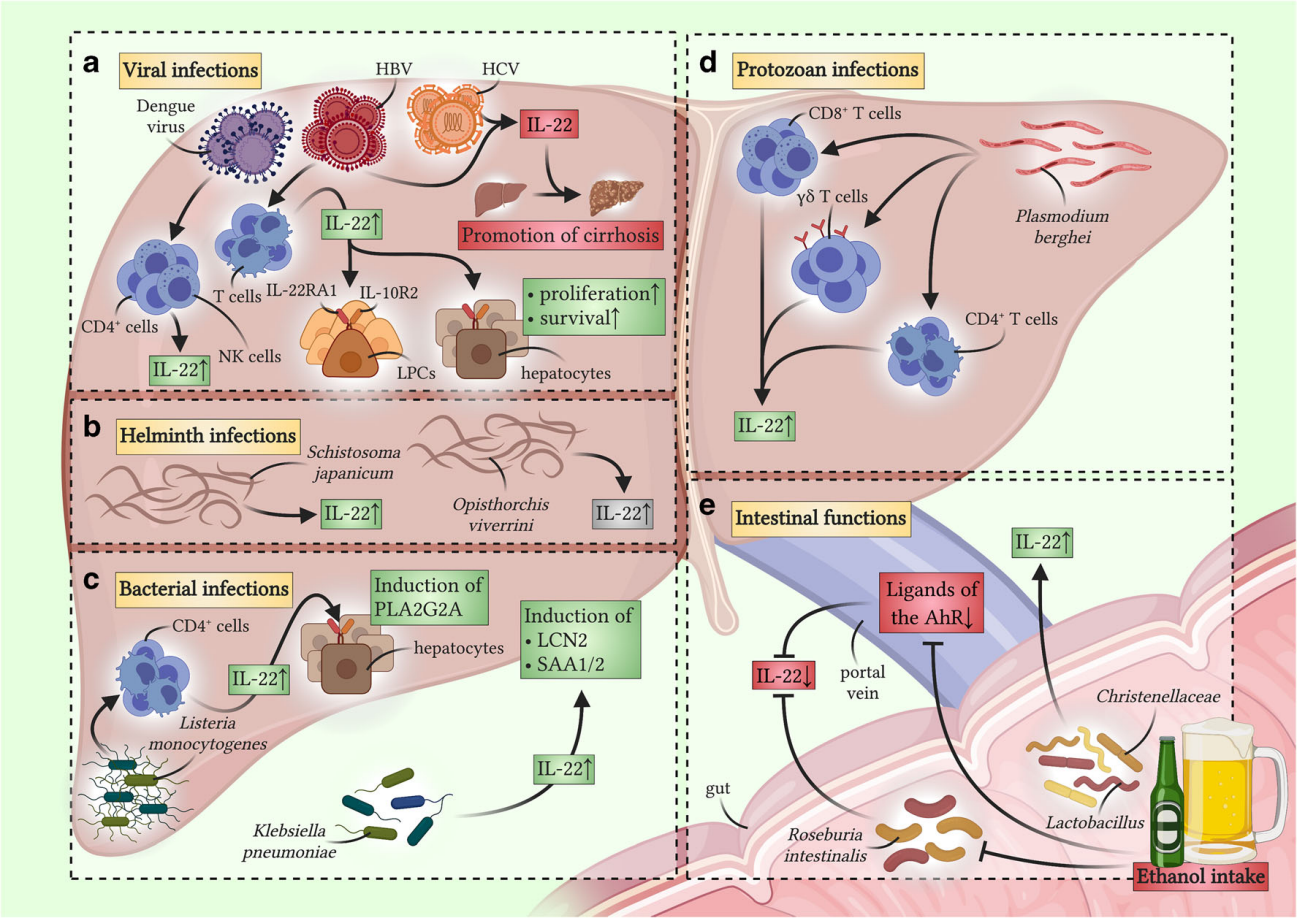
Functions of IL-22 during liver infections. Beneficial pro-inflammatory effects are displayed in green boxes, while pathogenic or harmful effects are depicted in red boxes. Unclear functions are depicted in grey. Generally, current literature suggests a strong protective effect of IL-22 during liver infections [46–48, 53, 55, 58, 60, 65]. (**a**) Induction of IL-22 by different viral liver infections. While Dengue virus 2 induces a protective production of IL-22 produced by NK cells and CD4^+^ T cells [60], HBV and HCV lead to a more ambiguous response. On the one hand, an HBV-infection can trigger a T-cell-based production of IL-22, which exerts protective effects such as promoting survival and proliferation on hepatocytes and liver progenitor cells [65]. On the other hand, infections with HBV and HCV can also cause pathogenic IL-22, promoting or enhancing liver cirrhosis [67–69]. (**b**) Role of IL-22 during helminth liver infections. Infection with *Schistosoma japonicum* can trigger the production of protective IL-22 [58]. Likewise, *Opisthorchis viverrini* infections can cause an upregulated IL-22 expression; however, its general function in this context is unclear [59]. (**c**) Role of IL-22 during bacterial liver infections. In the liver, *Listeria monocytogenes* can cause increased production of IL-22 by CD4^+^ T cells [47]. In this context, IL-22 exerts protective functions by inducing the upregulation of antimicrobial peptides in hepatocytes, such as PLA2G2A [47]. Furthermore, intraperitoneal infection with *Klebsiella pneumoniae* equally leads to an IL-22-dependant increase of antimicrobial substrates, such as LCN2 or SAA1/2 [48]. (**d**) Role of IL-22 during protozoan liver infections. Protozoans such as *Plasmodium berghei ANKA* primarily target erythrocytes but also affect other organs, especially the liver. They are known to lead to enhanced production of IL-22 by CD4^+^ T cells, CD8^+^ T cells, or γδ T cells [53–55, 57]. In this context, IL-22 exerts protective functions. (**e**) Role of intestinal homeostasis to mediate liver-relevant production of IL-22. For example, *Lactobacillus* and *Christenellaceae* can mediate IL-22 production that exerts protective effects in patients suffering from NASH [45]. Contrarily, regular ethanol intake decreases the abundance of *Roseburia intestinalis* [43] and ligands of the AhR [44] to dampen an otherwise protective IL-22 response. Functions of IL-22BP during liver infections remain largely elusive and, thus, are not depicted in this figure

To summarize, IL-22 has dual effects in chronic liver inflammations, while the role of IL-22 in acute liver infections is considered to be protective overall (Fig. 1 and Table 1). Regrettably, it can be assumed that only half of the picture is known in these infectious circumstances as there is a lack of information on the soluble inhibitor of IL-22, IL-22BP. This gap in our knowledge presents us with a growing challenge of understanding how IL-22 signaling can be targeted to combat these infections.

**Table 1:** Overall impact of IL-22 and IL-22BP in different liver infections

Group	Disease	IL-22	IL-22BP
Infection	Bacterial infections	Protective effects during *Listeria monocytogenes* infections [47]	Unknown effects
		Protective effects during *Klebsiella pneumoniae* infections [48]	
	Protozoan infections	Protective effects during *Plasmodium chabaudi* infections [55]	Unknown effects
		Protective effects during *Plasmodium berghei ANKA* infections [53]	
	Helminth infections	Possible protective effects during *Schistosoma japonicum* infections [58]	Possible pathogenic effects during *Schistosoma japonicum* infections [58]
	Viral infections	Protective effects during Dengue virus 2 infections [60]	Unknown effects
		Possible protective effects during hepatitis B virus infections [65]	
		Potential pathogenic effects during hepatitis B and hepatitis C virus infections [67–69]	

Summarized roles according to current literature

## IL-22 and IL-22BP in liver damage

### Alcoholic liver disease (ALD)

Alcoholic liver disease (ALD) describes an acquired form of liver damage due to excessive alcohol consumption, and approximately 35% of people with an excessive rate of alcohol intake develop ALD [70]. The influence of IL-22 on this disease has been thoroughly investigated. In line with its overly protective effects during acute infections, IL-22 possesses equally safeguarding roles here [71, 72]. In mouse models, administration of recombinant IL-22 and overexpression with an injection of an IL-22 adenovirus protected mice from liver pathology induced by chronic binge-feeding of ethanol [71, 72]. In human ALD, an increase of IL-22 produced by CD4^+^ T cells could be detected in peripheral blood [73]. Moreover, a high frequency of this IL-22-producing subset was associated with a better short-term outcome [73]. Another report could correlate IL-22 serum levels of ALD patients with scores describing the extent of liver cirrhosis and damage [74]. Comparable to many other causes of liver damage, IL-22 is often discussed to exert potent hepatocyte-protecting functions [75], such as preventing apoptosis in ethanol-damaged hepatocytes [76]. Thus, it is rather unsurprising that IL-22 was implied as a possible therapeutical treatment for ALD. Recently, a phase-2 study investigating the influence of a recombinant fusion protein of human IL-22-Fc dimer in ALD was completed [77]. Indeed, this drug, also termed F-652, decreased clinical scores and liver serum markers, increasing hopes for treating ALD in the near future.

In contrast to its functional counterpart, IL-22, little is known regarding the effects of IL-22BP in ALD. A recent study identified reduced levels of IL-22BP in patients suffering from alcoholic hepatitis, increasing the bioavailability of IL-22 [78]. However, this report equally found that low expression of IL-22BP was correlated with decreased survival rates [78]. It could be presumed that a permanent activation of the inflammasome dampens IL-22BP expression, leading to an overall dysregulation of the immune system independent of the IL-22/IL-22BP axis. Nonetheless, further studies are needed to decipher the precise role and implications of these immune mediators within this disease context.

### Toxic liver damage

Apart from liver damage induced by excessive ethanol intake, other substances are also known to cause hepatic inflammation and subsequent destruction. In a clinical context, drug-induced hepatotoxicity is a potent, widespread problem [79]. Thus, multiple mouse models have been developed using agents such as concanavalin A (ConA), carbon tetrachloride (CCl_4_), D-galactosamine in combination with lipopolysaccharide (Gal/LPS) or acetaminophen (APAP) to mimic certain aspects of toxic liver damage [80, 81]. In line with findings from other damage-associated models, IL-22 has been shown to exert a strong protective effect in many of these methods. First observations already underlined such functions in 2004, where depletion of IL-22 was associated with increased severity of ConA- and CCl_4_-induced hepatitis [82–84]. Later on, equal observations were made when exposing mice to Gal/LPS [85] while treating them with an IL-22 blocking antibody. With regard to APAP-induced liver damage, several studies could detect a beneficial effect of a short-term treatment with recombinant IL-22 [86, 87]. Although another report detected an equal protective effect of administered recombinant IL-22 during APAP hepatitis, the authors of this report found an increased susceptibility of IL-22TG mice to this method of hepatitis induction [88]. Interestingly, this observation was partially independent of STAT3-mediated downstream signaling [88]. In line with this last study, a further report revealed that IL-22BP-deficient mice displayed a higher susceptibility to APAP-mediated liver damage [89]. Since the production of IL-22 is not controlled in IL-22BP-deficient mice, it corroborates the hypothesis that a chronic, dysregulated production of IL-22 does more damage than good. However, there is still sufficient proof of a protective role of IL-22 in toxic liver damage when tightly regulated or only shortly overexpressed.

### Nonalcoholic steatohepatitis (NASH)

Another form of liver damage results from a long-term intake of high fat and high caloric food often associated with a Western diet [90]. This liver damage ultimately culminates in NASH, a disease that affects up to 6% of the citizens of the USA [90]. Due to the mainly protective functions in other inflammatory liver diseases, a spotlight was cast once more on the effect of IL-22 in this particular liver pathology. Unsurprisingly, serum levels of IL-22 were greatly upregulated in a murine model of NASH progression [91]. Furthermore, multiple mouse studies feeding mice a high-fat diet detected protective functions of this cytokine, ameliorating the overall disease progression [92–94]. In particular, mRNA levels of multiple regulators for lipogenesis were found to be reduced upon a 2-week lasting treatment with recombinant IL-22 when feeding mice a methionine-choline-deficient diet (MCDD) [92]. A more recent report could expand the suspected protective influences of IL-22 by demonstrating that this cytokine could likewise mediate protection in a neutrophil-driven model of NASH [94]. Here, the authors showed that by treating mice with an IL-22-Fc dimer, IL-22 was not only able to reduce lipogenesis and apoptosis but also reduce inflammation and oxidative stress via the specific induction of the anti-oxidative agent metallothionein [94]. Of note, IL-22-Fc treatment also proves beneficial in the treatment of other metabolic diseases, such as diabetes [95].

After considering what is known so far about this cytokine in liver pathologies, IL-22 treatment might be a suitable therapeutic regime for alleviating NASH. Apart from the approach being evaluated for ALD, where an IL-22-Fc dimer is used to ameliorate disease progression, other drug delivery techniques have also been considered. For example, another report recently developed a liver-specific nano-complex that delivered an IL-22-encoding gene specifically to the liver of mice [93]. Subsequently, this newly developed treatment was capable of inhibiting NASH progression [93]. However, next to the refinement of IL-22-overexpressing therapies for the treatment of liver pathologies, further research should be directed towards deciphering the influence of IL-22BP in this disease as a possible complementary therapeutic target for NASH.

### Ischemic liver damage

Finally, ischemic insults complete the list of the most common causes of liver damage. Ischemic insults in the liver arise either locally by an infarct, which may result from multiple factors, or globally by an intermittent reduction or complete lack of blood supply to this organ [96]. The latter also naturally occurs during liver surgery, especially during liver transplantation [97]. However, pro-longed hypoxemia of this delicate organ combined with a phase of rapid reperfusion leads to ischemia-reperfusion injury (IRI), which is associated with severe complications [97]. In this context, the roles of IL-22 and IL-22BP have been elucidated by two studies [89, 98].

The first study investigated the role of IL-22 in mediating the rejection of successfully transplanted livers in rats [98]. It was found that antibody-mediated inhibition of IL-22 led to increased liver damage during the early IRI-phase, possibly by an increase of apoptosis and reduced regeneration capacities in these rats [98]. However, rats were protected from an acute rejection after 7 days due to a decrease of pro-inflammatory mediators [98]. In line with the pathogenic long-term effects of IL-22 during liver transplantation in rats, the second study could corroborate the harmful effects of dysregulated IL-22 by using IL-22BP-deficient mice [89]. Strikingly, the authors already detected increased liver damage in IL-22BP-deficient mice 48 h after a successfully performed IRI [89], leading to the assumption that possible protective effects of IL-22 require a tight regulation by IL-22BP.

Furthermore, IL-22-deficient mice did not display a less severe phenotype than their wild-type counterparts during IRI, indicating that possible protective effects of IL-22 might be highly time-dependent and might not exist during the entire course of the disease. Moreover, this report found an increase of CD11b^+^ CD11c^+^ and CD11b^+^ Ly6C^high^ cells and an upregulated expression of C-X-C motif chemokine ligand 10 (CXCL10) and IFNα in IL-22BP-deficient mice [89]. Conversely, treatment with an antibody blocking CXCL10 ameliorated IRI-induced liver damage in IL-22BP-deficient mice, but not in the wild-type controls. Hence, this study suggested that overt IL-22 production might increase the expression of CXCL10, which in turn promoted tissue damage via the recruitment of pro-inflammatory immune subsets [89].

Conclusively, both studies investigating the roles of IL-22 and IL-22BP in ischemic liver damage corroborate what has been identified in toxic liver models. A consensus is that in acute phases of liver damage, a tightly regulated IL-22 exerts protective effects via different mechanisms. However, induction of prolonged or dysregulated IL-22 production might lead to a pathogenic outcome and increased tissue damage.

### Liver regeneration

The regenerative capacity of the liver is a central aspect of its functionality. Not only can small functional lesions be easily repaired by newly proliferating tissue, but it also allows for simultaneous surgical resections of multiple lobes of the liver without permanent impairments [99]. To this day, multiple studies depict IL-22 as an important player in mediating wound healing and tissue regeneration [22]. Likewise, IL-22 is well-known for promoting liver regeneration [100–104]. In 2010, an initial study provided the first proof of IL-22 representing an important engine for liver regeneration [100]. Using a model of partial hepatectomy in mice, the authors detected an upregulation of IL-22 in the serum of mice shortly after they underwent partial hepatectomy and also detected decreased proliferation in mice pretreated with an IL-22 blocking antibody [100]. Subsequently, increased levels of IL-22 were also detected in patients shortly after they had undergone major liver resection [101]. Interestingly, ILCs were found to constitute the major source of IL-22 during liver regeneration in mice and humans in this report [101].

Finally, IL-22 also enhances the regenerative capacity of pre-damaged livers. In two independent studies, mice injected with ConA before partial hepatectomy was performed exhibited an ameliorated outcome when the mice were treated with recombinant IL-22 or IL-22 fusion protein [102, 103]. Moreover, a protective effect of IL-22 could also be determined during a newly developed model for acute-on-chronic liver failure (ACLF), in which mice were given an acute high dose of CCl_4_ after having been treated with this agent for 8 weeks prior [104]. Strikingly, treatment with IL-22-Fc did not only ameliorate liver damage but also significantly reduced the blood bacterial load in this model, hinting at multiple beneficial targets of increased levels of IL-22 during liver regeneration. To summarize, while the role of IL-22BP during liver regeneration remains elusive, its counterpart, IL-22, exhibits a strong regenerative effect. With IL-22-Fc as a possible therapeutic agent, prognosis and survival rates of patients after extended liver surgery could be significantly increased.

### Liver fibrosis and cirrhosis

**Fig. 2:**
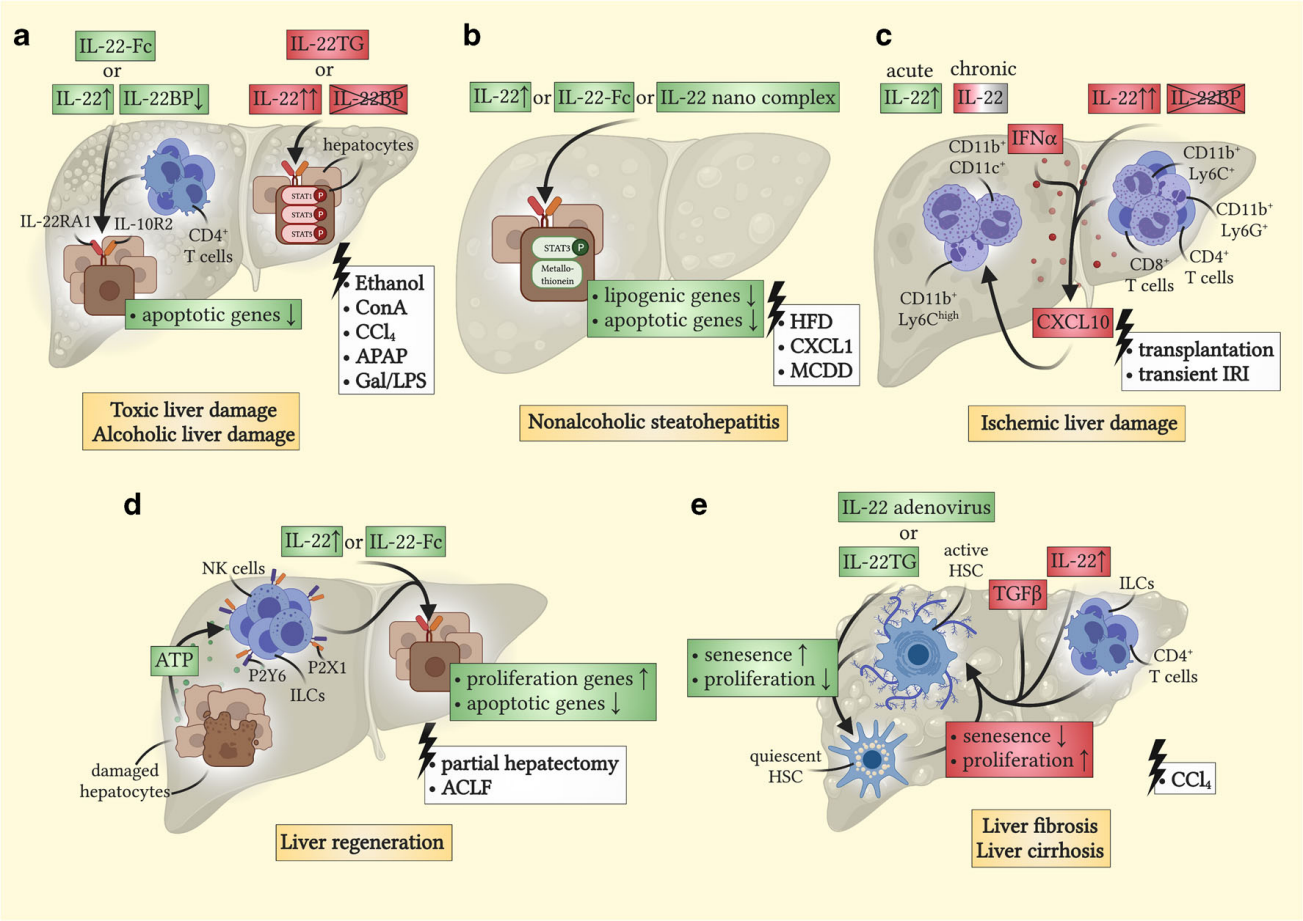
Functions of IL-22 and IL-22BP during different liver damage causes, regeneration, fibrosis, and cirrhosis. Beneficial pro-inflammatory effects are displayed in green boxes, while pathogenic or harmful effects are depicted in red boxes. Unclear functions are depicted in grey. (**a**) Roles of IL-22 and IL-22BP in toxic liver disease and ALD. Different mouse models were used to induce toxic liver damage, such as ethanol binge-feeding [71, 72] or applications of ConA [82–84], CCl_4_ [83], APAP [86–88], or Gal/LPS [84, 85]. In general, IL-22 is upregulated while IL-22BP is found downregulated in toxic liver damage [73, 74, 78]. Among other cellular sources, CD4^+^ T cells are known to produce IL-22 in toxic liver damage [73]. Possible protective effects of IL-22 and IL-22-Fc include reducing apoptotic genes in hepatocytes [75, 76]. However, overexpressed IL-22, typically found in IL-22TG mice, or uncontrolled IL-22 activity in IL-22BP-deficient mice causes pathogenic effects [88, 89]. These functions are partially STAT3-independent, and thus, might rely on phosphorylation of STAT1 or STAT5 [88]. (**b**) Role of IL-22 in NASH. Different mouse models were used to induce NASH, such as a high-fat diet (HFD) [93], a methionine-choline-deficient diet (MCDD) [92], or an overexpression of CXCL1 [94]. IL-22 expression is generally upregulated in NASH [91]. IL-22, IL-22-Fc, and nano complexes delivering an IL-22 encoding gene were shown to exert protective effects [92–94]. These effects include reducing lipogenic genes [92], apoptotic genes, and oxidative stress [94] in hepatocytes. (**c**) Roles of IL-22 and IL-22BP in ischemic liver damage. Different mouse models were used to induce ischemic liver damage, such as liver transplantation [98] or transient IRI [89]. While acutely upregulated IL-22 has protective effects in a model of rat liver transplantation [98], chronic IL-22 exposure might have no influence or even pathogenic functions [89, 98]. Dysregulated IL-22 production in the absence of IL-22BP might equally exert pathogenic roles [89]. Here, overt IL-22 is produced as a response to transient IRI-damage by different cell subsets, such as CD4^+^ T cells, CD8^+^ T cells, CD11b^+^ Ly6C^+^, and CD11b^+^ Ly6G^+^ cells [89]. Consequently, IFNα and CXCL10 expression are upregulated, which might lead to enhanced recruitment of pro-inflammatory and pathogenic cells such as CD11b^+^ CD11c^+^ and CD11b^+^ Ly6C^high^ cells [89]. (**d**) Role of IL-22 in liver regeneration. Different mouse models were used, such as partial hepatectomy [100] or ACLF [104]. IL-22, partially produced by NK cells or ILCs, and IL-22-Fc were demonstrated to exert protective effects in this context [100–104]. Mechanistically, damaged cells increase free adenosine triphosphate (ATP) levels, which can bind to P2X1 and P2Y6 expressed on many cells [101]. This mechanism triggers an increase in IL-22, which can exert its protective functions such as induction of proliferation genes and reduction of apoptotic genes on cells like hepatocytes [101]. (**e**) Role of IL-22 in liver fibrosis and liver cirrhosis. Mainly, CCl_4_-induced liver cirrhosis was used to induce liver cirrhosis in mice [106, 109]. On the one hand, overexpression of IL-22 by injection of adenovirus delivering an IL-22 encoding gene or using IL-22TG mice indicates a protective effect of IL-22 [106]. This effect is mediated by increasing senescence and decreasing proliferation in activated HSCs, one of the main mediators of liver fibrosis [106–108]. On the other hand, the lack of IL-22RA1 indicates a pathogenic role of physiological levels of IL-22 [109]. IL-22 produced by CD4^+^ T cells and ILCs combined with TGFβ can activate HSCs by decreasing their senescence and increasing their proliferation [109]

Untreated acute and chronic liver damage can result in liver fibrosis and cirrhosis if its regenerative capacity is exceeded. Mechanistically, liver tissue is progressively and irreversibly replaced by a fibrotic scar, which can be understood as a misguided immune response to chronic liver damage [105]. While many cells contribute to the synthesis of liver scar tissue, activated hepatic stellate cells (HSC) are considered to be the central contributors to fibrogenesis [105]. Thus, much effort has been directed towards targeting HSCs in an attempt to alleviate liver fibrosis and cirrhosis [105]. Once again, a potential silver lining in the treatment of liver cirrhosis might lie within the protective effects of IL-22 [106–108]. Contrastingly, several reports outlined a rather pathogenic function of IL-22 by promoting liver fibrosis and cirrhosis [68, 109, 110]. These recent observations are, to some degree, surprising, especially with regard to the strong protective functions of IL-22 in many acute and some chronic liver conditions (Fig. 2 and Table 2).

**Table 2:** Overall impact of IL-22 and IL-22BP in different causes of liver damage, regeneration, fibrosis, and cirrhosis

Group	Disease	IL-22	IL-22BP
Damage	Alcoholic liver disease	Protective effects during chronic binge feeding in mice [71, 72]	Unknown effects
		Protective effects determined by clinical studies [73, 74, 77]	
		Protective effects due to in vitro studies [76]	
	Toxic liver damage	Protective effects during ConA-induced hepatitis in mice [82–84]	Unknown effects
		Protective effects during CCl_4_-induced hepatitis in mice [83]	
		Protective effects during Gal/LPS-induced hepatitis in mice [84, 85]	
		Mainly protective effects during APAP-induced hepatitis in mice [86–88]	Potentially protective effects during APAP-induced hepatitis in mice [89]
		Partially pathogenic effects during APAP-induced hepatitis in mice [88]	
	Nonalcoholic steatohepatitis	Protective effects during MCDD mediated NASH in mice [92]	Unknown effects
		Protective effects during HFD mediated NASH in mice [93]	
		Protective effects during HFD and CXCL1 mediated NASH in mice [94]	
	Ischemic liver damage	Partially protective effects during short-term inhibition directly after liver transplantation in rats [98]	Protective effects during IRI-induced hepatitis in mice [89]
		Partially pathogenic effects during long-term inhibition after liver transplantation in rats [98]	
	Liver regeneration	Protective effects during partial hepatectomy in mice [100, 102, 103]	Unknown effects
		Protective effects during acute-on-chronic liver failure in mice [104]	
		Protective effects determined by clinical studies [101]	
	Liver fibrosis and cirrhosis	Partially protective effects during CCl_4_-induced fibrosis in mice [106]	Unknown effects
		Partially protective effects due to in vitro studies [106–108]	
		Partially pathogenic effects during CCl_4_-induced fibrosis in mice [109]	
		Partially pathogenic effects due to in vitro studies [68, 109]	

Summarized roles according to current literature

On the one hand, IL-22 can directly target HSCs to reduce their fibrotic potential [106–108]. HSCs express IL-10R2 and IL-22RA1 and, therefore, can respond to IL-22 in vitro and in vivo [106]. In vitro stimulation of HSCs with IL-22 led to increased senescence, a reduced activation [106], and a decreased proliferation [107, 108], thus reducing their pro-fibrogenic potential. Moreover, IL-22TG mice were protected from CCl_4_-induced liver fibrosis, as were mice pretreated with an adenovirus containing an IL-22 encoding gene [106].

On the other hand, a report published a contrasting observation describing an increased proliferation and decreased senescence of HSCs upon in vitro stimulation with IL-22 [68]. Contrary to previous studies, the authors used a different human HSC cell line that they claimed was more typical for chronic liver disease [68]. In line with this, primary human HSCs displayed a profibrotic potential when co-stimulated with IL-22 and TGFβ, while stimulation with IL-22 alone did not result in any effect [109]. Furthermore, this study observed that IL-22 actually enhanced liver fibrosis by inducing CCl_4_-mediated liver damage in IL-22RA1-deficient mice. Consequently, the observed effect could be reversed by treatment with either AhR- or RAR-related orphan receptor gamma t (RORγt) antagonists [109]. One possible reason for the different observations made could be the functional difference between IL-22TG mice overexpressing IL-22 and IL-22RA1-deficient mice unable to respond to IL-22. It can be argued that the observed anti-fibrotic effect in IL-22TG mice [106] might not represent the physiological spatiotemporal expression pattern of IL-22. Thus, it also leads to an activation of other cell subsets, such as hepatocytes, ultimately leading to a protective effect. In humans, levels of IL-22 are elevated in patients suffering from liver cirrhosis, and high levels of IL-22 are associated with a worsened prognosis [110, 111]. Whether this clinical finding might be seen as a further indicator of a pathogenic role of IL-22 or whether this cytokine is just induced by other pathogenic factors that lead to reduced survival is currently unclear. Independent of its role during liver fibrosis, IL-22 possesses a rather pathogenic function during the development of HCC, as discussed below. Thus, elevated levels of IL-22 as they are seen in liver cirrhosis could further increase the risk of suffering from an HCC in these patients, even if this cytokine might exert primarily protective effects during liver cirrhosis.

In conclusion, the current data might encourage speculation that physiological levels of IL-22 enhance liver fibrosis, while overt IL-22 expression might induce more protective functions during fibrosis. In this context, studies investigating the role of IL-22BP as a central regulator of the bioavailability of IL-22 are highly relevant, however, currently missing. Especially a blockade of IL-22BP to unleash possible protective features of high levels of IL-22 might prove beneficial in certain therapeutical settings. However, caution is advised since IL-22 can also favor the development of HCC, a risk that is greatly increased for patients with liver fibrosis or cirrhosis anyhow. Thus, more data are urgently needed to evaluate the therapeutic benefit of modifying the IL-22/IL-22BP axis in liver fibrosis and cirrhosis.

## IL-22 and IL-22BP in liver malignancies

### Hepatocellular carcinoma (HCC)

HCC describes a malignant transformation of hepatocytes that mostly arises from a pre-cirrhotic liver [112]. Thus, most risk factors for the development of HCC are comparable with the ones of liver cirrhosis, mainly consisting of chronic HBV or HCV disease, ALD, and NASH [112]. Since early-stage symptoms of HCC often range from vague to non-existing, regular sonographic control of the liver is recommended once a patient is diagnosed with liver cirrhosis [113]. While multiple therapies exist for the early stages of HCC, such as surgical resection, liver transplantation, radiofrequency ablation (RFA), or transcatheter arterial chemoembolization (TACE), therapeutic options for more advanced stages of HCC are still limited [114]. Nonetheless, two recent therapeutic developments might offer hope. First, the development of multi-kinase inhibitors such as sorafenib has slightly elevated the overall prognosis of many patients in recent years [115]. Second, multiple clinical trials have been carried out investigating the role of checkpoint inhibitors in modifying HCC progression [116]. Strikingly, monotherapy with nivolumab or pembrolizumab led to a significant reduction in tumor size even after the failure of sorafenib treatment, so that multiple phase-3 trials are currently ongoing investigating the effect of a combination of different checkpoint inhibitors as HCC therapy [116]. However, advanced HCC is still a fatal disease in most cases today and calls for the continuous development of new therapeutic regimes.

IL-22 is often described as a cytokine with different roles in some cancer entities [117, 118]. While data suggests a rather pro-tumorigenic effect of this cytokine in breast and lung cancer, its role in colorectal cancer, for example, is more dichotomous [117, 118]. In many tumor environments, IL-22 signaling affects both the proliferation and stemness of healthy epithelium and dysplastic epithelium, providing a possible explanation for its diverse effects [117, 118].

Concerning HCC, many studies detected an upregulation of IL-22 in the serum of patients suffering from HCC [119–122]. Likewise, an increased IL-22 production could be measured in the tissue of HCC in comparison to cirrhotic or healthy tissue controls [121–124]. Moreover, it was found that IL-22 levels were equally correlated to clinical stages of HCC [119, 121]. When analyzing the overall impact of IL-22 in HCC development, three independent studies implied a pathogenic role of this cytokine since high serum levels of IL-22 were associated with a worse prognosis and shorter disease-free survival [122, 125, 126]. Interestingly, a reverse finding was made in patients that underwent TACE treatment [127]. Here, patients with a detectable level of IL-22 had significantly higher survival than patients whose IL-22 could not be detected [127]. This might lead to the assumption that IL-22 possesses possible protective effects as well by promoting the regeneration of healthy liver tissue. This anti-tumorigenic role of IL-22 might solely be relevant in conditions in which only a few malignant cells are left at the cost of destroying a significant amount of healthy liver tissue. However, further research needs to address the specifics of this observed dichotomous effect in HCC.

The overall described pathogenic role of IL-22 during the development of HCC in patients could be equally reproduced in different mouse models [62, 119]. The authors of these two studies used the well-established HCC model injecting the carcinogen diethylnitrosamine (DEN) into mice. Both reports found either an increased tumor burden in IL-22TG mice [62] or a decreased amount of tumors in IL-22-deficient mice [119], corroborating the HCC-promoting effect of IL-22. When analyzing the source of IL-22, studies found both T_H_17 cells and T_H_22 cells to be a major source of this cytokine in the patient’s blood and in the cancerous tissue itself [121, 125]. Different mechanisms have been identified to induce IL-22 production from these cell subsets in HCC. First, in vitro analysis from paired blood and tumor samples of patients revealed that antigen-presenting cells, especially B7 homolog 1 (B7-H1) (also termed programmed death-ligand 1 [PD-L1]) expressing monocytes, were capable of inducing the differentiation of T_H_22 cells [125]. In line with this finding, in vivo treatment with an antibody blocking B7-H1 (PD-L1) could significantly reduce the tumor size of subcutaneously implanted murine HCC cells in this study [125]. Of note, this monocyte-induced differentiation was co-dependent on IL-1β, IL-6, transforming growth factor-beta (TGFβ), and IL-23 [125]. Elevated levels of IL-23 could be detected likewise in the tumor tissue [119] so that it seems likely that both antigen-presentation and the production of soluble factors such as cytokines contribute to the expansion of IL-22 producing cells in HCC. Moreover, a recent study suggested that necrotic lysates of dying hepatic cancer cells might be able to induce enhanced production of IL-22 by T_H_17 cells as well [128].

**Fig. 3:**
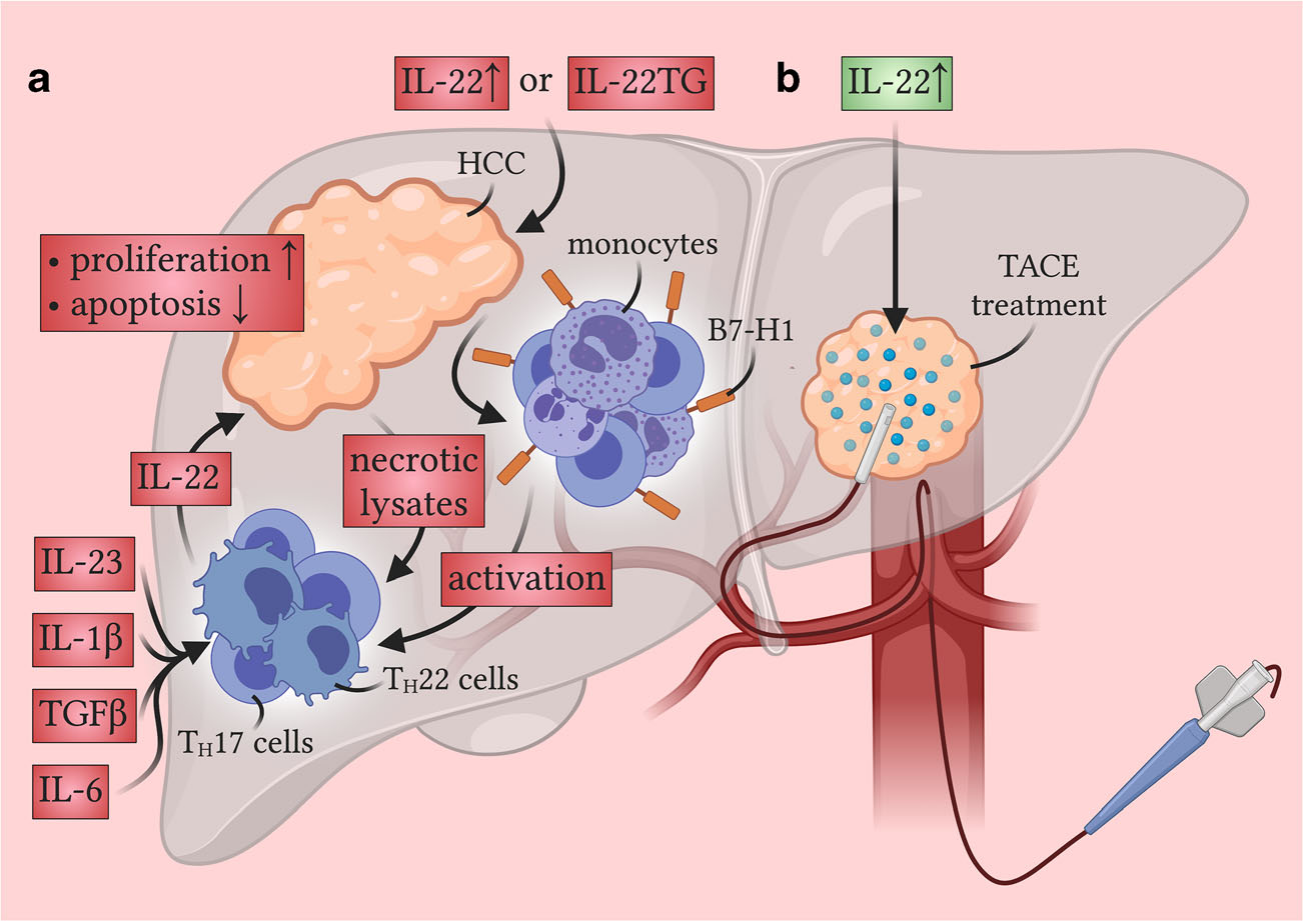
Functions of IL-22 during HCC development. Beneficial anti-tumorigenic effects are displayed in green boxes, while pathogenic pro-tumorigenic effects are depicted in red boxes. (**a**) Much evidence points to a pathogenic role of IL-22 in HCC development, as was found out by using IL-22-deficient or IL-22TG mice [62, 119]. IL-22, mostly derived from T_H_17 cells and T_H_22 cells [121, 125], directly acts on tumor cells by increasing their proliferation rate and decreasing their apoptotic potential [119, 125]. Different mechanisms can induce IL-22 production. Firstly, B7-H1 (PD-L1) expressing monocytes activated by cancerous tissue can induce T_H_22 cell differentiation [125]. Secondly, dying malignant hepatocytes can likewise induce T_H_22 cell expansion by secreting necrotic lysates [128]. Thirdly, other cytokines such as IL-1β, IL-6, IL-23, or TGFβ might enhance IL-22 production [119, 125]. (**b**) Interestingly, IL-22 seems to exert a partially protective role in HCC patients treated by TACE [127]

As described above, IL-22 exerts its downstream activity through the recruitment and phosphorylation of STAT3 [18]. Thus, an increase of phosphorylated STAT3 could be detected in tumoral tissue in the liver [119]. Additionally, in vitro observations proved that IL-22 mainly exerts its pro-tumorigenic effects by enhancing cell proliferation and inhibiting apoptosis of the tumor cells themselves [119, 125]. Although IL-22 likely possesses other cellular targets other than tumor cells, these have not been discovered so far. In conclusion, IL-22 derived from T_H_17 cells, T_H_22 cells, and potentially other sources can promote HCC progression through different mechanisms, mainly acting through the cancer cells (Fig. 3 and Table 3). However, further investigations are needed to unravel not only further targets of IL-22 but also the role of IL-22BP during liver carcinogenesis.

**Table 3:** Overall impact of IL-22 and IL-22BP in different malignant liver degenerations

Group	Disease	IL-22	IL-22BP
Malignant degeneration	Hepatocellular carcinoma	Pathogenic effects during HCC development [62, 119, 122, 125, 126]	Unknown effects
		Potential protective effects during TACE treatment [127]	
	Cholangiocarcinoma	Unknown effects	Unknown effects
	Liver metastasis	Unknown effects	Unknown effects

Summarized roles according to current literature

### Cholangiocarcinoma (CCA)

Another primary liver malignancy is CCA, originating from dysplasia of the bile duct’s epithelial cells. Although CCA only accounts for 15% of all primary liver tumors in its entirety, therapy of CCA still results in enormous challenges, often due to its late diagnosis [39]. Unfortunately, not much is known regarding the role of IL-22 during CCA and its diverse causes. A pioneering study that was also discussed extensively above investigated the impact of this cytokine in a liver fluke-dependent sub-entity of CCA for the first time. In brief, the authors showed that IL-22 expression was upregulated in peripheral blood of CCA patients and that production of IL-22 was especially enhanced in CD4^+^ T cells [59]. However, these findings need to be expanded before even a preliminary evaluation of IL-22 in CCA can be performed.

### Liver metastasis

Although the clinical implications of sufficient treatment of liver metastasis are undeniable, no study has yet investigated the functions of IL-22 or IL-22BP in this context. However, defining the role of these immune mediators in metastasis might be of utter importance to expand the treatment options of this highly lethal disease. Regarding the overly pathological role of IL-22 in many cancer entities [117, 118], it is quite likely that the fight against liver metastasis would equally benefit from an inhibition of IL-22. Thus, an investigation focusing on understanding the IL-22/IL-22BP axis in this area of cancer research is highly warranted.

## Outlook

Taken together, IL-22 possesses both protective and pathogenic traits during the course of different liver pathologies. Whether IL-22 acts as a friend or as a foe is highly dependent on the specific disease and the duration of IL-22 exposure, the latter being mainly regulated by its natural antagonist, IL-22BP. Conclusively, three possible treatment options arise from the above-discussed implications of these two immune mediators. Firstly, recombinant IL-22 might be used for treating toxic liver damage. Indeed, one recent phase-2 study reported reduced liver serum enzymes and liver-damage-associated scores in patients suffering from alcoholic hepatitis [77]. Since uncontrolled or chronic IL-22 production might lead to an undesirable effect as observed when treating IL-22BP-deficient and IL-22TG mice with APAP [88, 89], caution is advised. Secondly, therapeutic regimes could take advantage of an IL-22 blocking antibody in liver diseases in which IL-22 was shown to exhibit pathogenic effects, such as HCC. An example of this includes fezakinumab, which is already being evaluated for psoriasis treatment [129]. Thirdly, administration of recombinant IL-22BP or IL-22BP encoding messenger ribonucleic acid (mRNA) should equally be considered in patients suffering from HCC. The functionality of the latter approach was already demonstrated by a recent in vivo study, in which colorectal tumor growth could be successfully prevented by the delivery of mRNA in the host [130].

Undoubtedly, an encounter of IL-22 and IL-22BP will almost always result in an inseparable formation. Whether this mutual affection and the following fear of detachment have positive or negative effects is highly dependent on the context. On the one hand, and comparable to small children, observed separation anxiety is a physiological response needed to form a definitive ego distinct from the mother or prevent overt inflammation, respectively [5]. On the other hand, a chronic clinging to the mother over an extended period of time is often considered pathological, as is an increased binding of IL-22BP to IL-22 during chronic inflammatory diseases [30, 31]. Unfortunately, evidence documenting the direct pathogenic roles of IL-22BP in the liver is still scarce. Contrarily, it is well-documented that uncontrolled IL-22 expression in the absence of IL-22BP might lead to a different outcome than short-term treatment with IL-22 [88, 89]. Thus, a balanced relationship is needed in psychological and biological contexts to restrict separation anxiety to its physiological intended frame.

## Abbreviations

ACLF: Acute-on-chronic liver failure
AhR: Aryl hydrocarbon receptor
ALD: Alcoholic liver disease
APAP: Acetaminophen
ATP: Adenosine triphosphate
B7-H1: B7 homolog 1
BP: Binding protein
CCA: Cholangiocarcinoma
CCl_4_: Carbon tetrachloride
CD: Cluster of differentiation
ConA: Concanavalin A
CXCL10: C-X-C motif chemokine ligand 10
DC: Dendritic cell
DEN: Diethyl nitrosamine
DENV2: Dengue virus 2
FMT: Fecal microbiota transplant
Gal/LPS: D-galactosamine in combination with lipopolysaccharide
HBV: Hepatitis B virus
HCC: Hepatocellular carcinoma
HCV: Hepatitis C virus
HSC: Hepatic stellate cells
IL: Interleukin
IL-22TG: IL-22 transgenic
ILC3: Type 3 innate lymphoid cell
IL-TIF: Interleukin-10-related T cell-derived inducible factor
IFNα: Interferon-alpha
IFNγ: Interferon-gamma
IFNλ: Interferon-lambda
IRI: Ischemia-reperfusion injury
LCN2: Lipocalin 2
LSEC: Liver sinusoidal endothelial cell
MCDD: Methionine choline-deficient diet
mRNA: Messenger ribonucleic acid
NASH: Nonalcoholic steatohepatitis
NKT cell: Natural killer T cell
PD-L1: Programmed death-ligand 1
PLA2G2A: Phospholipase group II A
RORγt: RAR-related orphan receptor gamma t
RFA: Radiofrequency ablation
Reg3g: Regenerating islet-derived protein 3 gamma
SAA1/2: Serum amyloid A1 and A2
SHIV: Simian-human immunodeficiency virus
SNP: Single nucleotide polymorphism
STAT: Signal transducer and activator of transcription
TACE: Transcatheter arterial chemoembolization
TGFβ: Transforming growth factor-beta
T_H_17 cell: T helper 17 cell
T_H_22 cell: T helper 22 cell
